# Prevalence of temporomandibular disorder in children and adolescents with juvenile idiopathic arthritis – a Norwegian cross- sectional multicentre study

**DOI:** 10.1186/s12903-020-01234-z

**Published:** 2020-10-13

**Authors:** J. Fischer, M. S. Skeie, K. Rosendahl, K. Tylleskär, S. Lie, X.-Q. Shi, E. Grut Gil, L. Cetrelli, J. Halbig, L. von Wangenheim Marti, M. Rygg, P. Frid, P. Stoustrup, A. Rosèn

**Affiliations:** 1 Department of Clinical Dentistry, The Faculty of Medicine, University of Bergen, Årstadveien 19, N-5009 Bergen, Norway; 2 Center for Oral Health Services and Research of Middle-Norway (TkMidt), Trondheim, Norway; 3 Department of Radiology, University Hospital of North Norway, Tromsø, Norway; 4 UiT the Arctic University of North Norway, Tromsø, Norway; 5 Department of Pediatrics, Haukeland University Hospital, Bergen, Norway; 6 Department of Oral and Maxillofacial Radiology, Faculty of Odontology, University of Malmö, Malmö, Sweden; 7 Public Dental Service Competence Centre of Northern-Norway (TkNN), Tromsø, Norway; 8 Department of Clinical and Molecular Medicine, NTNU - Norwegian University of Science and Technology, Trondheim, Norway; 9 Department of Pediatrics, St. Olavs Hospital, Trondheim, Norway; 10 Department of Otorhinolaryngology, Division of Oral and Maxillofacial Surgery, University Hospital North Norway, Tromsø, Norway; 11 Department of Clinical Medicine, Faculty of Health Sciences, The Arctic University of Norway, Tromsø, Norway; 12 Section of Orthodontics, Faculty of Health Sciences, Aarhus University, Aarhus, Denmark; 13 Department of Oral and Maxillofacial Surgery, Haukeland University Hospital, Bergen, Norway

**Keywords:** Juvenile idiopathic arthritis, Temporomandibular joint arthritis, Temporomandibular disorder, Temporomandibular joint disease, Children and adolescents

## Abstract

**Background:**

Children and adolescents with juvenile idiopathic arthritis (JIA) may suffer pain from temporomandibular disorder (TMD). Still, routines for the assessment of temporomandibular joint (TMJ) pain in health and dental care are lacking. The aims of this study were to examine the prevalence of TMD in children and adolescents with JIA compared to their healthy peers and to investigate potential associations between JIA and TMD.

**Methods:**

This comparative cross-sectional study is part of a longitudinal multicentre study performed during 2015–2020, including 228 children and adolescents aged 4–16 years with a diagnosis of JIA according to the ILAR criteria. This particular substudy draws on a subset of data from the first study visit, including assessments of TMD as part of a broader oral health examination. Children and adolescents with JIA were matched with healthy controls according to gender, age, and centre site. Five calibrated examiners performed the clinical oral examinations according to a standardised protocol, including shortened versions of the diagnostic criteria for TMD (DC/TMD) and the TMJaw Recommendations for Clinical TMJ Assessment in Patients Diagnosed with JIA. Symptoms were recorded and followed by a clinical examination assessing the masticatory muscles and TMJs.

**Results:**

In our cohort of 221 participants with JIA and 221 healthy controls, 88 (39.8%) participants with JIA and 25 (11.3%) healthy controls presented with TMD based on symptoms and clinical signs. Painful TMD during the last 30 days was reported in 59 (26.7%) participants with JIA vs. 10 (5.0%) of the healthy controls (*p* <  0.001). Vertical unassisted jaw movement was lower in participants with JIA than in controls, with means of 46.2 mm vs. 49.0 mm, respectively (p <  0.001). Among participants with JIA, a higher proportion of those using synthetic disease-modifying antirheumatic-drugs and biologic disease-modifying antirheumatic-drugs presented with painful masticatory muscles and TMJs at palpation.

**Conclusion:**

Symptoms and clinical signs of TMD were seen in approximately half of the JIA patients compared to about one fourth of their healthy peers. Painful palpation to masticatory muscles and decreased vertical unassisted jaw movement were more frequent in participants with JIA than among healthy controls and should be part of both medical and dental routine examinations in patients with JIA.

## Background

Juvenile idiopathic arthritis (JIA) is currently the most common chronic rheumatic disease in children and adolescents [1, 2]. The International League of Associations of Rheumatology (ILAR) defines JIA as arthritis of unknown aetiology, starting before the age of 16 years with a duration of at least 6 weeks [3]. It encompasses seven categories, including systemic arthritis, oligoarthritis (persistent or extended), rheumatoid factor negative polyarthritis, rheumatoid factor positive polyarthritis, psoriatic arthritis, and enthesitis-related arthritis with different, though overlapping, characteristics. Cases that fit none or more than one of these categories are defined as undifferentiated arthritis. The burden of JIA is characterised by short and long-term functional disability and pain. Common features at presentation are morning stiffness, swelling of one or more joints, functional disturbances, and sometimes pain. The reported prevalence is around 1–2 cases per 1000 children, with girls more frequently affected than boys [1, 2].

Temporomandibular disorder (TMD), known as an umbrella or collective term for muscle pain and jaw dysfunction, covers a heterogeneous group of conditions [4]. TMD is associated with various clinical signs and symptoms involving the masticatory muscles, teeth, tongue, temporomandibular joint (TMJ), and/or their supportive tissues [5–7]. Changes in motor behaviour caused by musculoskeletal pain and pain-related movement disorders reflect sustained pain perception. In two recent studies from Western Norway [8, 9], the prevalence of painful TMD among otherwise healthy adolescents was reported to be around 7% based on self-reported pain screening questionnaires adopted by Nilsson and colleagues [10]. In the study by Graue and colleagues [9], the prevalence of TMD was 11.9% when using the Diagnostic Criteria for Temporomandibular Disorders (DC/TMD). In all three studies, females were more frequently affected than males.

In children and adolescents with JIA, the reported figures are substantially higher. Previous studies of children and adolescents with JIA reported a broad spectrum of TMD prevalence ranging between 39 and 87% [11–13], depending on the study designs and the sample size. Therefore, it is a need to reinforce the evidence with a relatively high number of samples and give insights to the effects of medication in JIA participants when they are examined by palpation of masticatory muscles and TMJ.

Previous studies revealed that children and adolescents, irrespective of their JIA category, are prone to develop TMJ arthritis [11, 14]. Also, younger children with JIA might suffer pain from TMJs caused by inflammation and/or destructive changes, by muscular tensions from the surrounding muscles as a component of TMD, or by a combination of the two [12]. Symptoms indicating TMJ arthritis include decreased mouth opening and/or ear ache and pain during eating, chewing, or yawning [15–17]. At present, there are no precise clinical or imaging markers for active TMJ arthritis [18, 19]. As for TMJ involvement, several studies have shown that even significant deformities may be undiagnosed due to a lack of symptoms or clinical findings [16, 20–22]. In younger children, the clinical assessment of painful TMD symptoms might be biased by indirect input from their parents.

The aims of the present study were to examine the prevalence of TMD in children and adolescents with JIA compared to their healthy peers and to investigate potential associations between JIA and TMD.

## Methods

### Study design and participants

This cross-sectional study was part of a longitudinal multicentre study, the NorJIA study, performed during 2015–2020 and including 228 children and adolescents. Inclusion criteria were a diagnosis of JIA according to the ILAR [3] and age 4–16 years. In the exclusion criteria, absent of written informed consent or major medical comorbidities such as congenital facial anomalies, skeletal dysplasia or malignancies were excluded.

This particular substudy (2015–2018), using a matched comparative cross-sectional design, drew on a subset of data from the first study visit, including assessments of TMD as part of a broader oral health examination. Children and adolescents were matched (1:1) with healthy controls according to gender, age, and centre site. The healthy controls were recruited from seven different Public Dental Service clinics representing both rural and urban areas in the western, middle, and northern parts of Norway. The sample size estimate was based on a Swedish study reporting a TMD prevalence of 26% in children with JIA [23], and a sample size of 296 was required for a precision of 5% with a 95% confidence interval.

### Data collection

At the study visits, children and adolescents with JIA were examined by experienced paediatric rheumatologists at Haukeland University Hospital in Bergen, University Hospital of North Norway in Tromsø, and St. Olavs University Hospital in Trondheim. Registered data included background characteristics in terms of age at disease onset, disease category, disease status on the day of the examination, a thorough joint examination, blood tests, and validated measures for patient-reported disability, general body pain, and health assessments. Furthermore, the applied dose was according to the international recommendations, while duration varied significantly, with or without combination with other medication. However, detailed drug history concerning duration and doses was not available in the study database. Both children and adolescents with JIA and controls underwent a thorough clinical oral examination performed by experienced dentists, including a TMD assessment.

### TMD screening and assessment

The assessment procedures were standardised and were based on two shortened versions of the diagnostic tools “Axis I Clinical Examination for DC/TMD” [20] and the self-assessment questionnaire “TMJaw Recommendations for Clinical TMJ Assessment in Patients Diagnosed with JIA” [21]. The latter was used to enhance the operational specification of DC/TMD due to the fact that the DC/TMD tool alone is reported to show weak validity for TMJ assessment, e.g. disc displacement diagnosis (low sensitivity) and degenerative joint disease diagnosis (low sensitivity and specificity) [20].

Prior to and during the study period, calibration sessions for the five participating oral examiners were performed, including four calibration exercises according to procedures previously described by our research team [22]. Further details on the calibration results are presented in Supplementary Tables S1 and S2.

### Variables and outcomes

The demographic variables were age, gender, JIA categories, and medication status. The subjective symptom outcomes were TMD pain in the last 30 days (n, %) reported by the participants or the parents. The examining dentists also registered how many of the individuals expressed pain during jaw movement in the clinical examination (n, %). The clinical outcomes included vertical and lateral unassisted jaw movements (mm), pain upon palpation of the masticatory muscles and the TMJ (n), and if the TMJ disc was clicking in a painful manner (n).

### Statistical methods

Two-way mixed intraclass correlation coefficient (ICC) and percent agreement were used for calibration measurements. Differences between groups were tested using Chi-square statistics or a two-sample t-test as appropriate. All statistical tests were performed using SPSS version 25 (IBM, Chicago, IL). The level of statistical significance was set at 5% (*p* ≤ 0.05).

### Ethical considerations

The study was approved by the regional ethics committee (2012/542/REK vest). Written informed consents were obtained from all parents and/or participants as appropriate. The study was registered at ClinicalTrials.gov (No: NCT03904459).

## Results

**Fig. 1:**
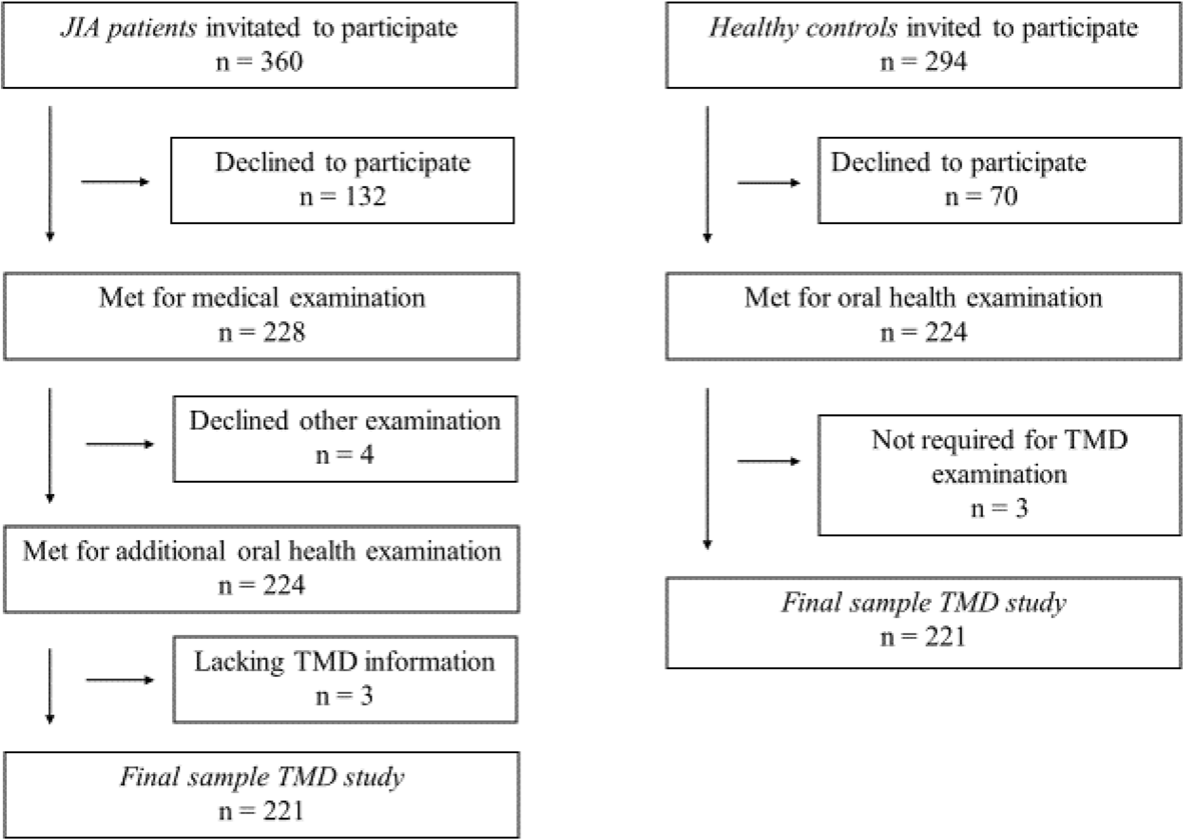
Flow chart of patients and healthy individuals included in the study

A total of 360 children and adolescents with JIA were eligible for the main study, of whom 228 accepted the invitation to participate, yielding a response rate of 63.3%. The acceptance rate for healthy controls was 224/294 (76.2%). The mean age for participants with JIA and healthy controls was 12.0 years (SD 3.17 and 3.21, respectively) (*p* = 0.98), and the mean age of the 228 participants with JIA was higher than for the 132 eligible patients that did not participate at 12.0 years vs. 10.5 years (SD 3.16 and 3.5, respectively) (*p* <  0.001). The proportion of girls with JIA was also higher than among the 132 patients not participating (59.2% vs. 58.3%, *p* = 0.027). Among the 228 participating children with JIA, 224 underwent an oral examination and 221 underwent the TMD assessment and were thus included in the present substudy (Fig. 1).

Of the 221 children with JIA, 132 were girls (59.7%), the median age at disease onset was 6.1 years (IQR 8.1, 2.3–10.4), the median age at the study visit by paediatric rheumatologists at the hospital was 12.7 years (IQR (5.3, 9.4–14.7), and the median disease duration was 4.6 years (IQR 5.7, 2.6–8.3) (Table 1). Oligoarticular JIA was the most common category and was seen in 98 of 221 patients (44.3%) with 77 having persistent oligoarticular disease and 21 having extended disease. In total, 146 of the 221 patients (66.1%) had on-going medication with synthetic disease-modifying antirheumatic-drugs (sDMARDs) and/or biologic disease-modifying antirheumatic-drugs (bDMARDs).

**Table 1:** Clinical characteristics of participants with Juvenile idiopathic arthritis (JIA) in relation to temporomandibular disorder (TMD)

	Total cohort	TMD	No TMD
	*n* = 221	*n* = 88	*n* = 133
	Value	Value	Value
Girls, n (%)	132 (59.7)	61 (69.3)	71 (53.4)
Age at onset, median (IQR)	6.1 (8.1, 2.3–10.4)	6.8 (8.4, 0.7–14.2)	5.2 (7.2, 0.9–14.7)
Age at visit by paediatric rheumatologists at the hospital, median (IQR)	12.7 (5.3, 9.4–14.7)	13.1 (3.3, 5.2–16.1)	11.7 (6.5, 4.8–16.5)
Disease duration, median (IQR)	4.6 (5.7, 2.6–8.3)	4.6 (6.0, 0.2–14.2)	4.6 (5.5, 0.2–14.7)
**JIA categories, n (%)**			
Oligoarthritis persistent	77 (34.8)	27 (30.7)	50 (37.6)
Oligoarthritis extended	21 (9.5)	11 (12.5)	10 (7.5)
Systemic arthritis	7 (3.2)	2 (2.3)	5 (3.8)
RF negative polyarthritis	49 (22.2)	17 (19.3)	32 (24.1)
RF positive polyarthritis	4 (1.8)	2 (2.3)	2 (1.5)
Psoriatic arthritis	9 (4.1)	6 (6.8)	3 (2.3)
Enthesitis-related arthritis	23 (10.4)	9 (10.2)	14 (10.5)
Undifferentiated JIA	31 (14.0)	14 (15.9)	17 (12.8)
**Ongoing medication, n (%)**			
No DMARDs	75 (33.9)	26 (11.8)	49 (22.2)
sDMARDs*	60 (27.1)	23 (10.4)	37 (16.7)
bDMARDs**	86 (38.9)	39 (17.6)	47 (21.3)

*JIA* Juvenile idiopathic arthritis, *TMD* Temporomandibular disorder

*sDMARDs = Synthetic disease-modifying antirheumatic- drugs; methotrexate, mykofenolatmofetil, **bDMARDs = Biologic disease-modifying antirheumatic-drugs; etanercept, infliximab, adalimumab, tocilizumab, abatacept, certolizumab, golimumab

### Clinical oral examination

**Fig. 2:**
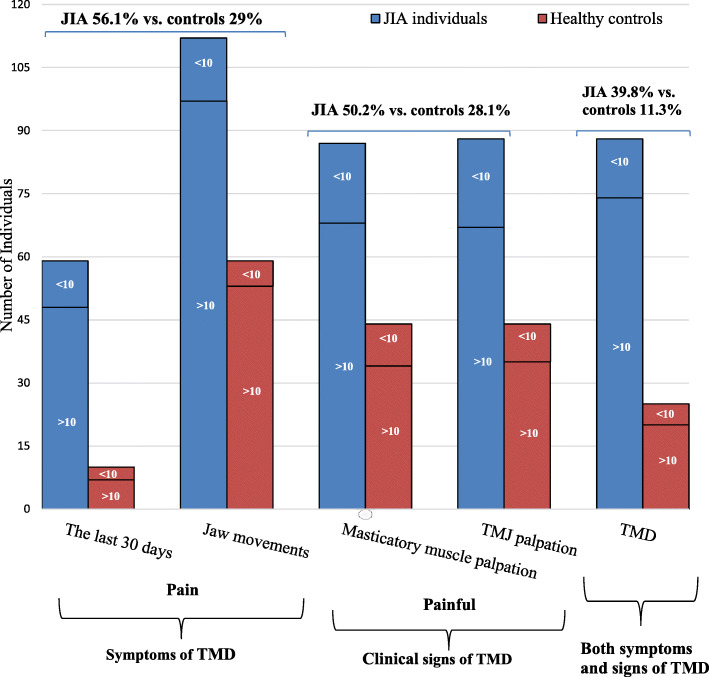
Prevalence of TMD in children and adolescents with JIA vs. healthy peers, ≥ 10 years and < 10 years of age, 1) symptoms: pain the last 30 days and pain with jaw movements; 2) clinical signs: pain upon palpation of the masticatory muscles and TMJ, and 3) a combination of symptoms (1) and clinical signs (2)

Taking into consideration that self-reported pain is a combination of parent-reported and participant-reported pain outcome, self-reported pain in the jaws during the last 30 days was reported in 59 (26.7%, 44 girls) participants with JIA vs. 10 (5%, 8 girls) in healthy controls (*p* <  0.001). Pain during jaw movements at the clinical examination was reported in 112 (51%, 67 girls) participants with JIA vs. 59 (26.8%, 34 girls) in healthy controls (*p* <  0.001) (Fig. 2), ranging from 28.6 to 50% in the different JIA categories (Table 1). No statistically significant differences in the presence of TMD according to JIA categories were found (*p* = 0.848) (results not shown).

The clinical examination revealed that the mean vertical unassisted jaw movement was lower for participants with JIA than for controls, 46.2 mm vs. 49.0 mm, respectively (*p* <  0.001) (Table 2). A total of 88 (39.8%, 61 girls) participants with JIA and 25 (11.3%, 17 girls) healthy controls had both symptoms and clinical signs of TMD (Fig. 2). When assessing the jaw muscles and TMJ, 111 (50.2%, 75 girls) participants with JIA had both painful masticatory muscles and TMJs on palpation vs. 62 (28.2%, 39 girls) of the healthy controls (p <  0.001) (Table 3). A higher proportion of participants on current sDMARDs and/or bDMARDs treatment presented with painful masticatory muscles and TMJ at palpation compared to participants with no biologic treatment (Table 4).

**Table 2:** Jaw movement in 221 participants with JIA compared to controls

Jaw movement	n	JIA	n	Healthy controls	*p*-value^*^
		mm		mm	
		mean (SD)		mean (SD)	
Vertical unassisted	221	46.2 (7.1)	221	49.0 (6.7)	< 0.001
Lateral to the right side	215	9.7 (2.2)	220	9.8 (2.1)	0.408
Lateral to the left side	211	9.7 (2.4)	219	10.1 (2.0)	0.077

*JIA* Juvenile idiopathic arthritis, *SD* Standard deviation. ^*^Student’s t-test

**Table 3:** Pain on palpation and painful clicking in participants with JIA and controls

Clinical signs	n	JIA, n (%)	n	Healthy controls n (%)	*p*-value^*^
Painful palpation in masticatory muscles & TMJ	220	111 (50.5)	220	62 (28.2)	< 0.001
Painful palpation in masticatory muscle	217	87 (40.1)	218	44 (20.2)	< 0.001
Painful palpation at the TMJ lateral pole	220	64 (29.1)	219	29 (13.2)	< 0.001
Painful palpation around TMJ lateral pole	220	75 (34.1)	219	33 (15.1)	< 0.001
Painful clicking	221	13 (5.9)	221	2 (0.9)	0.0041

*JIA* Juvenile idiopathic arthritis, *TMJ* Temporomandibular joints. *Chi-square test

**Table 4:** Clinical signs and pain at palpation according to DMARDs in 221 participants with JIA

	Current sDMARDs and/or bDMARDs		No current sDMARDs and/or bDMARDs		*p*-value^*^
	n	n (%)	n	n (%)	
Vertical unassisted jaw movement (> 40 mm)	146	113 (77.4)	75	62 (82.7)	0.361
Painful palpation to masticatory muscles & TMJ	145	80 (72.1)	75	31 (41.3)	0.052
Painful palpation to masticatory muscles	143	62 (43.4)	74	25 (33.8)	0.173
Painful palpation at the TMJ lateral pole	145	49 (33.8)	75	15 (20.0)	0.033
Painful palpation at the TMJ around lateral pole	145	56 (38.6)	75	19 (25.3)	0.049

*sDMARDs* Synthetic disease-modifying antirheumatic-drugs; methotrexate, mykofenolatmofetil, *bDMARDs* Biologic disease-modifying antirheumatic- drugs; etanercept, infliximab, adalimumab, tocilizumab, abatacept, certolizumab, golimumab, *TMJ* temporomandibular joint, * Chi-square test

Among participants with JIA, there were no significant differences in vertical unassisted jaw movement according to medication, with a mean of 46.4 mm (SD 7.1) in the JIA group and 45.8 mm (SD 7.1) among those not using DMARDs (*p* = 0.986) (results not shown). However, in both groups, more than half of the participants had a vertical unassisted jaw movement of more than 40 mm. The proportion without this medication treatment was slightly higher (82.7%) compared to those on current sDMARDs and/or bDMARDs (77.4%).

## Discussion

We have shown using a comparative cross-sectional multicentre design that around one third of the participants with JIA in this cohort had TMD. Half of children and adolescents with JIA reported pain during jaw movements and pain on palpation of the masticatory muscles and TMJs as compared to one fourth of their healthy peers, palpatory pain was associated with sDMARDs and bDMARDs treatment, and children and adolescents with JIA had a significantly lower mean vertical unassisted jaw movement. Moreover, TMJ-related clinical signs and vertical unassisted jaw movement ≤40 mm had the highest association in the JIA group.

The reported prevalence of TMD in children with JIA varies between 38 and 83% according to the definitions and methods of ascertainment used, to the cohort examined, and to differences in populations [15, 24–27]. Ferraz and colleagues, in their study of 15 children with JIA ranging in age from 6 to 28 years (mean age 16.3 years), reported a high prevalence of 83%. Still, they did not describe the method of ascertainment, i.e., whether the figures were based on self-reporting or on clinical examination [28]. A previous study from Rongo and colleagues based on 50 participants with JIA aged 9–16 years found a prevalence of TMJ damage from 100 joints to be 74% as assessed by MRI [25]. Others have reported a prevalence of 55% based on a questionnaire [29] and of 72% based on clinical signs [24]. However, none of those studies were based on the research diagnostic criteria RDC/TMD, and the children were older than those in our study. In contrast, a longitudinal study by Zwir et al., including 75 children (mean age 12.4 years), revealed a prevalence of 38% based on symptoms and 47% based on clinical examination [30]. Their results are in line with ours.

In our study, the prevalence of TMD, either based on symptoms or clinical signs, in the healthy peers, were quite high at 28 and 29%, respectively. This was higher than in earlier studies among adolescents reported by Graue and colleagues (7 and 12%, respectively) and Østensjø and colleagues (7%) [8, 9]. Studies from Finland and Brazil confirm our results with a high prevalence of TMD in the normal population. Vierola et al. [26] reported a TMD prevalence of 35% (mean age 7.9 years) and de Paiva Bertoli reported a TMD prevalence of 34% (mean age 11.0 years) [27]. The difference in TMD prevalence in the normal population of children and adolescents is probably due to the use of different diagnostic tools, different numbers of participants, different ages of the studied populations, different countries, and different study designs. In studies from Norway, Graue and colleagues [9] used two screening questions for pain related to TMD [10] and DC/TMD [20] for symptoms and clinical signs in a population of 210 children and adolescents aged 12–19 years. Østensjø et al. [8] used the same two screening questions of TMD symptoms [10] for screening a population of 560 adolescents aged 13–19 years. Then a modified RDC/TMD examination [31] was used for those who answered yes to 1) having pain in the temples, face, TMJ, or jaws once a week or more and 2) having pain once a week or more when opening the mouth wide or chewing. The Finnish group [26] used the RDC/TMD [31] for clinical signs in 483 children aged 6–8 years, and the Brazilian group [27] used the American Academy of Orofacial Pain [32] form for screening and the RDC/TMD [31] for clinical examination in a population of 934 individuals aged 10–14 years. Thus it is clear that it can be challenging to get an exact figure on the prevalence of TMD in the normal population. A previous meta-analysis conducted by da Silva and colleagues showed the overall prevalence of intra-articular joint disorder to be 16% [33].

In our study, approximately half of the JIA subjects had clinical findings consistent with TMD, with no differences according to JIA category. Because the numbers for three of the categories – systemic arthritis, rheumatoid factor positive polyarthritis, and psoriatic arthritis – were relatively low, these results should be interpreted with caution.

The sensitivity and specificity of the clinical orofacial examination in relation to TMJ has been debated because displacement of the disc, although eliciting a clicking sound, might be asymptomatic [34–36]. Based on the DC/TMD criteria, asymptomatic TMJ clicking is still defined as TMD. However, several studies have shown that pain-free clicking represents a normal variant, typically seen in girls during puberty [15]. Recently, a clinical examination protocol for JIA was developed by the Temporomandibular Joint Juvenile Arthritis Working Group (TMJaw). This examination protocol focuses on three general items, namely TMJ symptoms, TMJ dysfunction, and dentofacial deformity in JIA, and it shows acceptable reliability and validity [7].

We found, in accordance with other studies, that the TMJ area and the masseter muscle region were common locations for pain in JIA [29]. However, a recent study from Koos and colleagues reported a lower frequency of masticatory pain on palpation [15], and Kristensen and colleagues stated that masticatory pain complaints could develop over time [37]. In the present study, more than half of the participants with JIA showed clinical signs in the TMJ region and the masseter region, and more than one-fourth of the participants with JIA had TMD. A longitudinal multicentre approach might elucidate the development of masticatory muscle pain, as Kristensen and colleagues have suggested [37].

The vertical unassisted jaw movement has been widely used as a valid marker for TMJ arthritis [38]. We showed that participants with JIA had lower vertical unassisted movements compared to their healthy peers, but the differences were relatively small, thus questioning its clinical significance. Viewed differently, for children and adolescents aged < 11 years, the cut-off value of 40 mm was within the range of normal vertical jaw movement [39]. Further, our findings suggest that lateral movement did not differ significantly between the two groups, which is in line with the results of Twilt and colleagues [40] and Küseler and colleagues [19]. In the latter study of 15 children with JIA with a mean age of 12 years, the recorded decreased lateral movements were ≤ 5 mm with no significant relevance [19].

We found no statistically significant differences in the presence of TMD according to JIA categories. However, we found a significantly higher occurrence of clinical signs in participants with JIA currently on DMARDs medication (whether synthetic or biologic) compared to those not taking such medication. A high risk of developing clinical signs of TMD was associated with a severe disease course, as indicated by the use of DMARDs.

The strengths of this study are the relatively large number of participants, in which the study groups were well matched, and the meticulous standardisation of the clinical TMJ assessment performed prior to and during the study period. However, the large number of participants should not hide the fact that we are dealing with an underpowered sample size that was lacking 75 participants. An additional limitation is that the overall response rate of 63%, although considered acceptable, might have influenced the results because the group that did not participate was, on average, slightly younger and had a somewhat lower proportion of girls. Also, the shortened version of the DC/TMD used in this study is not directly comparable with studies having used the full DC/TMD score. In the present study, children and adolescents with JIA with TMD involvement were defined based on clinical examination, and both self-reported and parent-reported pain. Further studies will focus on the role of imaging on the diagnosis of TMJ arthritis in children and adolescents with JIA. Clinical orofacial examination may not be reliable for diagnosing disc displacement without reduction [5]. Imaging diagnosis is particularly important in JIA with non-symptomatic TMJ involvement because hard tissue loss in the condyle might hinder the growth of the mandible and subsequently affect chewing function and cause aesthetic problems [15].

## Conclusion

Symptoms or clinical signs of TMD were seen in approximately half of the participants with JIA compared to about one fourth of their healthy peers. Painful palpation of masticatory muscles and decreased vertical unassisted jaw movement are more frequent in children with JIA than in healthy controls and should be part of both medical and dental routine examinations in the follow-up of JIA.

## Supplementary information

**Additional file 1 Table S1**. Reliability tests (using intraclass correlation coefficients) between “a reference” and the examiners.

**Additional file 2 Table S2**. Percent agreement values between “a reference” and the examiners.

## Data Availability

The datasets used and analysed in the current study are available from the corresponding author on reasonable request.

## Abbreviations

DC/TMD: Diagnostic criteria for temporomandibular disorder
bDMARDs: Biological disease-modifying antirheumatic drugs
DMARDs: Disease-modifying antirheumatic drugs
sDMARDs: Synthetic disease-modifying antirheumatic drugs
ILAR: International League of Associations of Rheumatology
JIA: Juvenile idiopathic arthritis
RDC/TMD: Research diagnostic criteria for TMD
TMD: Temporomandibular disorder
TMJ: Temporomandibular joint
