# Supplementary material for: Prevalence of temporomandibular disorder in children and adolescents with juvenile idiopathic arthritis – a Norwegian cross- sectional multicentre study
Source: BMC Oral Health. 2020 Oct 13;20:282. doi: 10.1186/s12903-020-01234-z (PMC7557069; doi:10.1186/s12903-020-01234-z)
Supplement: Supplementary file 1 — Additional file 1 Table S1. Reliability tests (using intraclass correlation coefficients) between “a reference” and the examiners. [file 12903_2020_1234_MOESM1_ESM.pdf]

Supplementary Table S1. Reliability tests between “a reference” and the examiners.

Reliability (intraclass correlation coefficients (ICCs)) for opening, lateral, and protrusive movements (mm). Test 1 (2015 Jan) shows ICC values between “a reference” and the examiner who examined the first participants included in the study. Test 2 (2015 Sep), Test 3 (2017 Feb), and Test 4 (2017 Nov) are all based on ICC values between “a reference” and other examiners. The ICC values reported are average measurements. For ICC interpretation, see https://www.ncbi.nlm.nih.gov/pmc/articles/PMC4913118/.

| | Test 1 | Test 2 | Test 3 | Test 4 |
|---|---|---|---|---|
| **Opening movements** | | | | |
| Pain-free opening | 0.85 (0.39,0.96) | 0.96 (0.98, 0.97) | 0.87 (0.84, 0.90) | 0.67 (-2.14, 0.96) |
| Max. unassisted opening | 0.93 (0.73, 0.98) | 0.99 (0.98, 0.99) | 0.92 (0.91, 0.94) | 0.97 (0.68, 0.99) |
| Max. assisted opening | 0.97 (0.87, 0.99) | 0.99 (0.98, 0.99) | 0.92 (0.91, 0.94) | 0.88 (-0.16, 0.99) |
| **Lateral at both sites and protrusive movements** | | | | |
| Right lateral | 0.78 (-0.09, 0.96) | 0.69 (0.43, 0.85) | 0.89 (0.85, 0.92) | 0.95 (0.53, 0.99) |
| Left lateral | 0.83 (0.31, 0.96) | 0.46 (0.34, 0.55) | 0.91 (0.89, 0.93) | 0.95 (0.52, 0.99) |
| Protrusion | 0.85 (0.38, 0.96) | 0.87 (0.84,0.90 | 0.88 (0.83, 0.93) | 0.96 (0.62, 0.99) |
