# Supplementary material for: Prevalence of temporomandibular disorder in children and adolescents with juvenile idiopathic arthritis – a Norwegian cross- sectional multicentre study
Source: BMC Oral Health. 2020 Oct 13;20:282. doi: 10.1186/s12903-020-01234-z (PMC7557069; doi:10.1186/s12903-020-01234-z)
Supplement: Supplementary file 2 — Additional file 2 Table S2. Percent agreement values between “a reference” and the examiners. [file 12903_2020_1234_MOESM2_ESM.pdf]

Supplementary Table S2. Percent agreement values between “a reference” and the examiners

Test 1 (2015 Jan) shows percent agreement values between “a reference” and the examiner who examined the first participants included in the study. Test 2 (2015 Sep), Test 3 (2017 Feb), and Test 4 (2017 Nov) are all based on percent agreement values between “a reference” and other examiners.

		Test 1		Test 2		Test 3		Test 4	
		Total	n (%)	Total	n (%)	Total	n (%)	Total	n (%)
M. temporalis[1]									
	Posterior	20	19 (95)	19.3	18.0 (93.3)	16.0	14.5 (90.9)	10	9 (90)
	Middle	20	18 (90)	19.0	16.3 (85.9)	16.0	14.0 (87.5)	10	10 (100)
	Anterior	20	20 (100)	18.0	14.3 (79.6)	16.0	13.5 (84.4)	10	6 (60)
M. masseter[2]									
	Origin	20	20 (100)	19.7	14.7 (74.6)	15.0	14.0 (93.3)	10	8 (80)
	Body	20	19 (90)	19.7	15.7 (79.7)	15.0	11.0 (73.3)	10	10 (100)
	Insertion	20	20 (100)	18	12.7 (70.6)	15.0	12.5 (83.3)	10	9 (90)
Lateral pol (both sides)									
		20	20 (100)	20	14.7 (73.5)	16.0	14.5 (90.6)	10	7 (70)
Around lateral pol (both sides)		20	20 (100)	20	14.3 (71.5)	16.0	10.0 (62.5)	10	9 (90)

[1]3 vertical zones together (both sides). [2]3 horizontal zones together (both sides).
